# Analysis of Microbial Community in the Archaeological Ruins of Liangzhu City and Study on Protective Materials

**DOI:** 10.3389/fmicb.2020.00684

**Published:** 2020-04-15

**Authors:** Mingliang Sun, Fengyu Zhang, Xinduo Huang, Yeqing Han, Nan Jiang, Biao Cui, Qingling Guo, Mengyue Kong, Lin Song, Jiao Pan

**Affiliations:** ^1^Ministry of Education Key Laboratory of Molecular Microbiology and Technology, Department of Microbiology, Nankai University, Tianjin, China; ^2^Institute of Applied Ecology, Chinese Academy of Sciences, Shenyang, China; ^3^Zhejiang Provincial Institute of Cultural Relics and Archaeology, Hangzhou, China; ^4^Hangzhou Liangzhu Archaeological Site Monitoring and Management Center, Hangzhou, China; ^5^Key Laboratory of Bioactive Materials, Ministry of Education, Department of Biochemistry and Molecular Biology, Nankai University, Tianjin, China

**Keywords:** the Archaeological Ruins of Liangzhu City, microflora, biodegradation, soil properties, protective materials

## Abstract

This study aims to provide a reference for the protection of the Archaeological Ruins of Liangzhu City. As a basis for the further preservation of these cultural relics, it is essential to analyze the microflora colonizing these soil objects. To do that, samples with microbial characteristics were obtained and analyzed by SEM and metagenomic sequencing to reveal the constitute of the microflora. We investigated the biodegradation of the protective material-epoxy resin by microorganisms in the Archaeological Ruins of Liangzhu City, and found that they would interact with each other, which would affect the performance of the epoxy resin. The specific mechanism of action requires further investigations. We evaluated the effect of ethyl orthosilicate on soil properties. Interestingly, we found that excess ethyl orthosilicate added to the soil of the Archaeological Ruins of Liangzhu City will cause a change in particle size and allowed the soil to condense in the laboratory. This indicates that the large use of orthosilicate may lead to intensified soil weathering, which in turn will cause soil erosion.

## Introduction

The Archaeological Ruins of Liangzhu City are a group of late Neolithic sites, 5300-4000 years old, covering a total area of approximately 34 square kilometers. They have rich connotations and include more than 50 sites centered on the Mojiaoshan site. Among these sites, anti-mountain tombs, the Yaoshan altar and the Mojiaoshan earth pyramid are the most important. Among the ruins, numerous villages, cemeteries, altars and other relics have been found. A large number of exquisite jade ritual objects are the most distinctive of the unearthed objects. The discovery of these relics has led the Archaeological Ruins of Liangzhu City to become one of the largest scales and level areas to be confirmed in the 5000-year history of Chinese civilization.

As places where cultural relics are preserved, earthen sites are an important component of cultural heritage demonstrating the unique spiritual value, vitality and creativity of a nation (Rodrigues et al., 2002). Protecting and utilizing earthen sites well can promote and protect national unity and enhance national confidence and cohesion. These sites have important far-reaching significance (Mathy, 2016). Earthen sites are widely distributed around the world, including the Dacunhe site from the Yangshao period (AIAA, 2014), the Yin site from the Xia and Shang period, the Luoyang site from the Sui and Tang dynasties and the Han and Tang dynasties, the Archaeological Ruins of Liangzhu City, the Great Wall site and Mayan sites in South America (Hammond et al., 1995; Martens et al., 2005). Soil sites can be divided into natural soil and artificial soil sites according to the soil texture, mainly composed of quartz, clay minerals, kaolinite and so on (Markus et al., 2007). Based on the immovable characteristics of earthen sites, workers responsible for cultural relic protection mainly choose appropriate reinforcement materials to infiltrate and reinforce them and protect the original sites. Reinforcement materials can be divided into organic materials (such as epoxy resin, or ethyl orthosilicate), inorganic-organic composite materials and inorganic materials (such as high-modulus potassium silicate (PS) or calcium hydroxide). Biodegradation refers to any irreversible change in the properties of a material caused by microorganisms and/or organisms belonging to different populations of systems (Valente, 1987). Biodegradable materials are those easily absorbed by microorganisms such as fungi and bacteria due to their chemical structure. Biodegradable materials can be organic natural or synthetic compounds or inorganic compounds. Natural polymers are very susceptible to biodegradation. For example, the degradation of cellulose has been extensively studied. Millions of tons of synthetic resin are produced annually worldwide for use as adhesives, coatings, inks, fabrics, and the like. Synthetic resins have been used by artists in their artwork, for example, as adhesives, or by protective personnel for protective treatments, for example, as a cement or protectant. Synthetic resins are generally less prone to chemical, physical, and biological degradation than other organic products, but many articles in the scientific literature (Boars, 1970; Eggins et al., 1971; Valente, 1987; Albertsson and Karlsson, 1993; Nakajima-Kambe et al., 1999) and cultural heritage protection literature claim that microorganisms can degrade synthetic resins. The biodegradability of a synthetic resin can be tested in different ways (Yang et al., 2008). The current standard methods for assessing the antimicrobial properties of synthetic resins are ASTM G21-96 (2002), “Standard Practices for Determining Antifungal Properties of Synthetic Polymeric Materials,” and ASTM G29-96 (2002), “Standard Practices.” In China, some cultural relics workers have begun to realize that biodegradability should be considered in the selection of reinforcement materials. Overseas, some researchers have carried out research on the antifungal and antialgal properties of reinforcement materials (Walchli and Zinkernagel, 1966; Dolezel, 1967).

In a previous study of the Archaeological Ruins of Liangzhu City (Fasi et al., 2014), our team found that there were abundant microorganisms in some areas, including algae, fungi, and bacteria. Additionally, some scientific researchers use more protective materials for earthen sites, and some protective materials have been invaded by microorganisms in other sites. Hence, the durability of materials is challenged, making it particularly important to test the microbial degradation of protective materials. In this study, epoxy resins were preliminarily tested as protective materials, and the effects of the use of tetraethyl orthosilicate (which is the top choice among more recent protective materials) as a protective material on soil were studied. This work lays a solid foundation for the study of biodegradability mechanisms and provides a reference for the protection of earthen sites.

## Materials and Methods

### Description of the Studied Site

**FIGURE 1:**
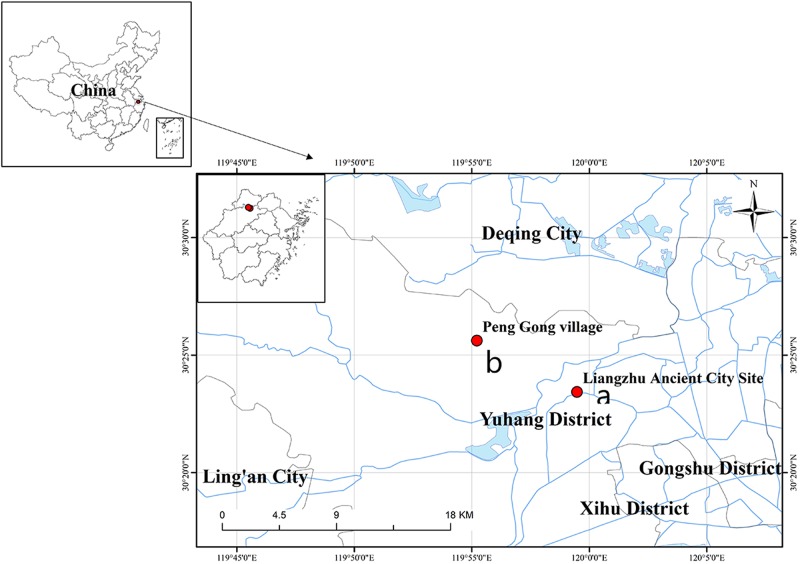
Location of the Archaeological Ruins of Liangzhu City in Zhejiang Heze, China. **(a)** Location of the South Wall sampling point. **(b)** Location of the Tiger Ridge sampling point. The map was drawn using ArcGIS v.10.2 (www.esri.com/arcgis).

The sampling points in this study included two locations: Tiger Ridge (LHL) and South Wall (NCQ). The Tiger Ridge sampling site is located at the Tiger Ridge Dam site of PengGong Village, Pingyao Town, Yuhang District, Hangzhou City, Zhejiang Province (Figure 1a), and the South Wall site is located in Liangzhu Ancient City Site Park, Pingyao Town, Yuhang District, Hangzhou City, Zhejiang Province (Figure 1b).

### Media

To isolate microorganisms from the soil samples from the Archaeological Ruins of Liangzhu City, we prepared two kinds of media: malt extract agar (MEA) medium supplemented with 50 mg/mL chloramphenicol to avoid bacterial growth and potato dextrose agar (PDA) medium. The potato dextrose agar (PDA) medium was used for susceptibility testing. Potato dextrose (PD) medium supplemented was used for the degradation testing of protective materials.

### Sampling

(I) Samples for microscopic analysis were obtained using double-sided carbon adhesive tape. In brief, a strip of carbon adhesive tape was gently placed on surfaces at the two sampling locations (Tiger Hill and South Wall). Three samples were taken from different locations at Tiger Ridge and South Wall, resulting in six samples that were finally taken to the laboratory. The collected samples were used for scanning electron microscopy (SEM).

(II) At the same sampling points, we used a sterilization key to collect 500 g soil samples for macro-genome sequencing and isolation of fungi, which were transported to the laboratory in an ice box for subsequent analysis.

(III) Samples for microscopic analysis were obtained using Scotch tape. The collected samples were used for optical microscopy.

### Microscopic Analysis

Scanning electron microscopy (SEM) was used to observe the microbial samples that adhered to the conductive adhesive and analyze the microbial species on the surface of the sites. The microbial samples were observed by optical microscopy, and the microbial species on the surfaces of the sites were analyzed.

### Dominant Fungal Identification and Isolation

Pure culture of isolate was fostered on a PDA plate, lasting 5 days at 28°C before DNA extraction, which was conducted through CTAB (Abdellaoui et al., 2011). Fungal 28S rRNA gene sequences were augmented by means of LR0R/LR7 primers (Kruys et al., 2015). ITS1-5.8S rRNA-ITS2 fungal gene sequences were augmented by means of ITS1/ITS4 primers. Sequencing of products of PCR were performed by GENEWIZ (Beijing, China), and the National Center for Biotechnology Information (NCBI) made analysis for sequential identity in BLAST program and the GenBank database.

### DNA Extraction and PCR Amplification

Extraction of the gross genomic DNA was carried out in soil through the MoBio PowerSoil^®^ DNA Isolation Kit (MO BIO Laboratories, Inc., CA, United States) in compliance with the manufacturer’s protocol.

Bacteria of 16S rRNA gene V4 area and fungi ITS1 area got augmented adopting consensus primers 515F/806R and ITS5–1737F/ITS2-2043R for prokaryotes and fungi, with a distinctive bar code placed on each sample (Lu et al., 2013; Ding et al., 2015). All PCR was conducted by means of Phusion^®^ High-Fidelity PCR Master Mix with GC Buffer (New England Biolabs, United Kingdom). The reaction mixture of 50 μL was amplified, containing 25 μL of master mix (2X), a 0.5 μM final concentration of forward and reverse primers, 10 ng of template DNA and nuclease-free water to 50 μL. The condition of PCR was set to 98°C for 1 min, and thirty cycles of 10 s at 98°C later, 30 s at 50°C for 16S rRNA genetic augmentation or 55°C for ITS regional augmentation, and 30 s at 72°C, extending 5 min at 72°C in the end. To visually verify the amplification of PCR, a volume equaling 1X the mounting buffer (consisting of SYBR green) and the PCR product was mounted onto a sepharose gel of 2%. A specimen, with an amplifying band from 400 to 450 bp, was selected to carry out further analysis. PCR products from various samples were gathered in equimolar amounts. Afterward, purification of the mixture of PCR product was proceeded by a Qiagen Gel Extraction Kit (Qiagen, Germany).

### Protective Material Degradation Experiment

**FIGURE 2:**
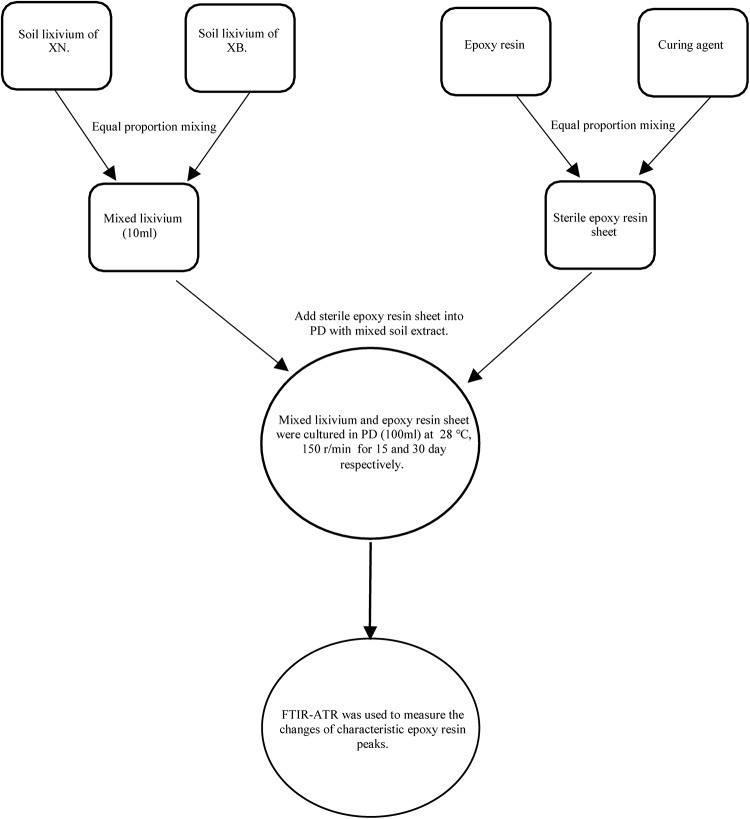
Flow chart of protective materials degradation experiment.

According to the literature, the protection of cultural heritage has become increasingly important. A basic aspect of protection work is to protect cultural relics in the later stage and avoid redestruction. In these later protection activities, many polymer protection materials are needed, such as acrylamide, pure acrylic emulsion, synthetic resin, epoxy, PS, B72, and ethyl orthosilicate (Cappitelli et al., 2004). The protective material will age and be damaged during use. Thus, we performed an experiment to investigate whether microorganisms are related to the aging damage of the protective material. We used epoxy resin as the protective material in this experiment (Pangallo et al., 2015). We evenly mixed the epoxy resin with the curing agent in a 1:1 ratio. To make a mold, we waited until the mixture solidified and obtained 1 ^∗^ 1 cm pieces of the film; we chose the four fungal isolates, NCQ2-2, NCQ2-3, NCQ3-1, and NCQ1-4, as the experimental strains. We mixed the four strains, and because these strains came from the South Wall site, we selected soil samples from the southwest and northwest corner of the South Wall to prepare a bacterial suspension. We used the method of shaking. Three parallel samples were selected for each group, and a control group was established. The sterilized epoxy resin material was added to the culture medium, and culturing was carried out for 30 or 60 days. Finally, we used Fourier transform infrared spectroscopy (FTIR-ATR) (Bruker TENSOR II) to characterize the changes in the material groups (Zafeiropoulos et al., 2003) (Figure 2).

### Soil Analysis Using Protective Materials

**FIGURE 3:**
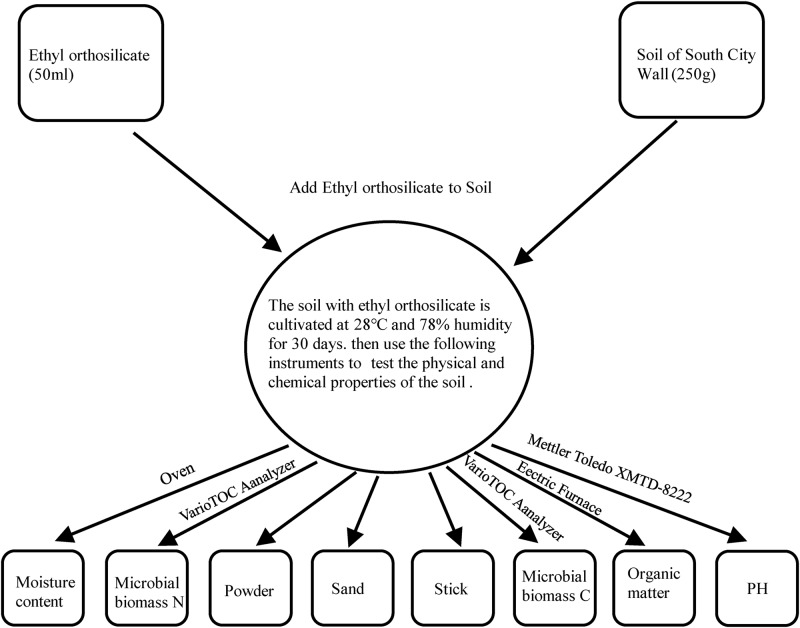
Flow chart of Soil analysis using protective materials.

In the protection activities performed at the Archaeological Ruins of Liangzhu City, many protective organic polymer materials such as ethyl orthosilicate were used, giving rise to the question of whether these materials cause damage to the soil ecosystem when they are used. Based on the above discussion, we collected some soil samples to simulate the environmental state of the Archaeological Ruins of Liangzhu City, and we selected ethyl orthosilicate as a protective material and added it to the soil to observe the changes in various properties of the soil over a 1-month cycle. The methodology was as follows: Collect two samples from the northwest and southwest corner of the South Wall of the Archaeological Ruins of Liangzhu City. From the above experiments, it was found that the isolated fungi mainly came from the samples from the South wall. For further analysis, two samples (XB, XN) were obtained from two sampling points on the South wall. There were two sets of experimental samples, with each group containing three parallel samples, and 250 g of soil per sample was placed in the medium. Then, 50 mL of tetraethyl orthosilicate was added, and 250 g of each of XN and XB was used as a control group. A total of 12 samples were examined. The above samples were placed in an incubator in which the temperature and humidity were controlled as much as possible in the local environment. After 1 month of culture, appropriate instruments (PH: Mettler Toledo XMTD-8222; microbial C, N: VarioTOC Analyzer, Elementar, Germany) (Šlepetieneė et al., 2010); H_2_O%: Oven; organic matter: electric furnace were used to detect PH, microbial C and N, H_2_O%, and organic matter levels and other properties (Figure 3).

### Nucleotide Sequence Accession

Nucleotide Sequence Accession Number The nucleotide sequences of strains have been deposited in the DDBJ/GenBank/EMBL database under the accession numbers MN509056-MN509073 for fungal ITS sequences. The raw sequencing data could be downloaded at the NCBI Sequence Read Archive (SRA) with the study accession number PRJNA573470.

## Results

### Microscopic Observation

**FIGURE 4:**
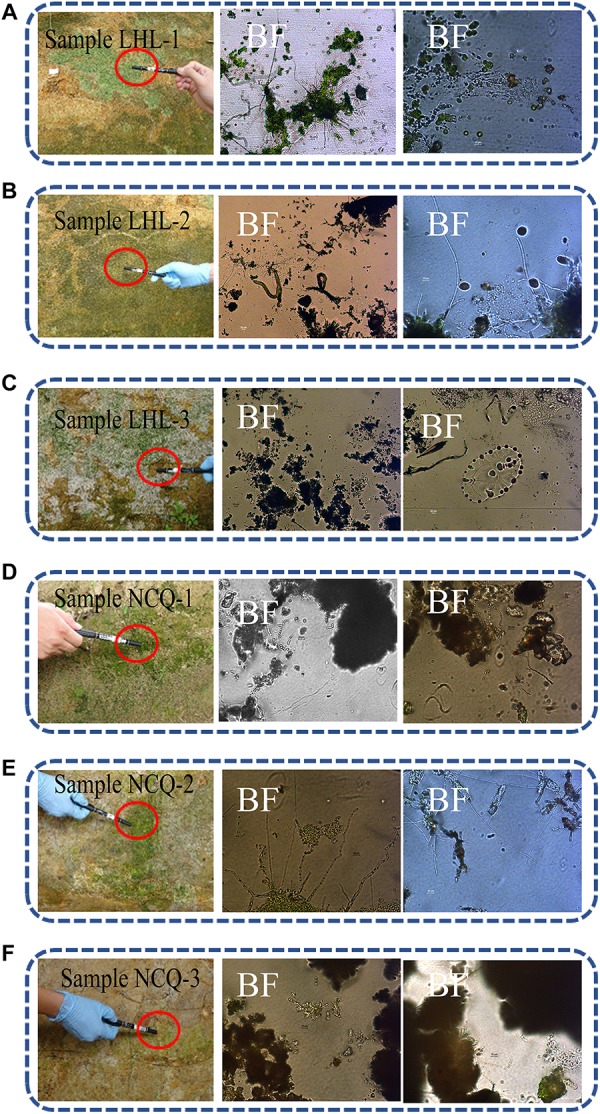
Sampling sites for microbiological analyses on the surfaces of six objects showing visible microbial contamination. **(A–F)** Details of samples LHL-1, LHL-2, LHL3, NCQ-1, NCQ-2 and NCQ-3. Visible signs of biofilm were present on the six samples; BF, bright field. In the bright field, a large number of green tissue structures, insect traps, beaded fungal hyphal structures and algae were observed.

Optical microscopy observations of the adhesive tape samples revealed the presence of fungal structures and algal structures. Algae can consist of one or a few cells, and many cells aggregate into tissue-like structures. The filaments can be branched or unbranched. Some algae are single-celled flagellates, while others aggregate into colonies. A large number of green tissue structures were observed in the LHL-1 and LHL-3 samples by optical microscopy, with the fungal hyphae separated; insect traps were observed in the LHL-2 samples, which were generally formed by fungal hyphae. These structures are exploited by fungi to capture nematodes; a beaded fungal hyphal structure was observed in NCQ-1, NCQ-2, and NCQ-3 samples (Figure 4).

**FIGURE 5:**
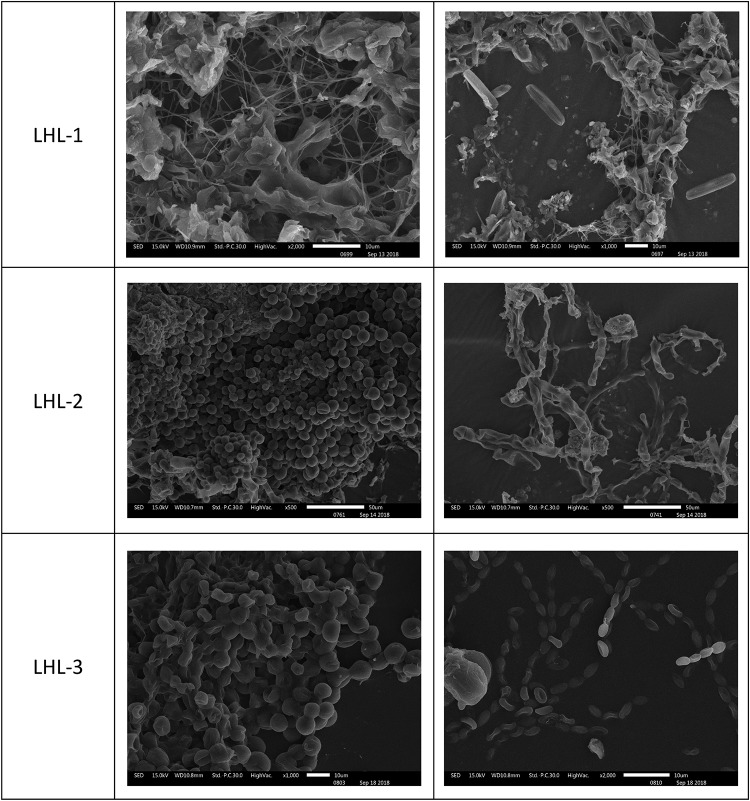
Scanning electron microscopy (SEM) images of a large number of hyphae and cells with a structure similar to algae observed in the sample.

**FIGURE 6:**
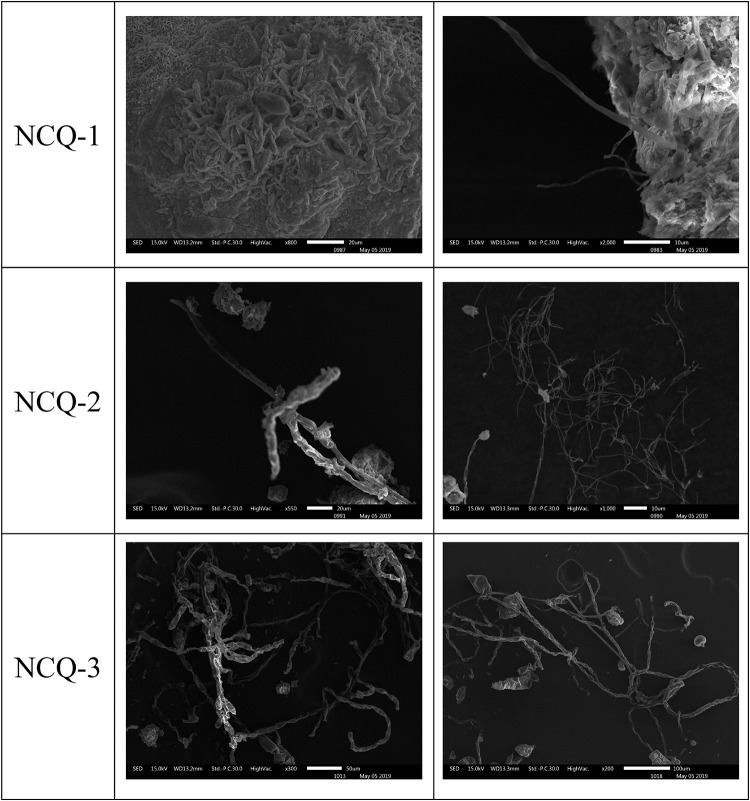
Scanning electron microscopy (SEM) images of a large number of hyphae and cells with a structure similar to algae observed on the sample.

The microscopic samples on the conductive adhesive were observed by scanning electron microscopy to analyze the microbial species present on the surfaces at the sites. The scanning electron microscopy results for LHL-1, LHL-2, and LHL-3 are shown in Figure 5. The structural features of typical fungal hyphae and algal cells were present in the samples. Electron micrographs of the LHL-1 samples showed a large number of hyphae on the surfaces at the site, with a cell structure similar to algae. In the scanning electron microscopy image from the South wall, we can see that there are a lot of algal cells as well as analogs of fungal hyphae. Images from NCQ-1 show the spores of fungi and some analogs of fungal hyphae. In the images from NCQ-2 and NCQ-3, various analogs of algal cells can be observed (Figure 6).

### Microbial Community Analyses

**FIGURE 7:**
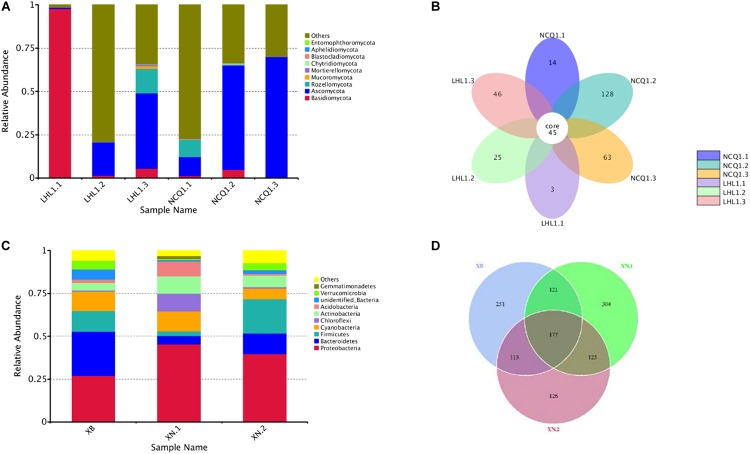
Relative abundance of the thirty most abundant microbial phyla and Venn diagrams showing shared OTU diversity among the samples. **(A)** The relative abundance for each sample is shown as a proportion of 100%. Fungal phyla are colored according to the legend on the right. **(B)** Fungal OTUs. **(C)** The relative abundance for each sample is shown as a proportion of 100%. Bacterial phyla are colored according to the legend on the right. **(D)** Bacterial OTUs.

A total of 431,056 fungal community reads were acquired with the filtration of low quality reads, chimeric sequences and trim adapter, bar codes, as well as primer. Ten fungal phyla were present in the three samples (Figure 7A). In different samples, the relevant richness of phyla varied a lot. Among the samples from Tiger Ridge, *Basidiomycota* is taken as the phylum of highest richness, with a proportion of 97.70% of the Tiger Ridge reads. A summary of the relevant richness of the fungal community at the genus level is shown in Table 1. The genera of fungi varied in samples. Of the 10 richest fungi taxa, *Lepidostroma, Penicillium, Aspergillus, Fusarium* existed in total specimens, while *Lepidostroma* proved to be genus with the largest abundance in the Tiger Ridge sample, accounting for between 1.47 and 97.66% of the Tiger Ridge sample, with a mean abundance of 33.85%. *Lepidostroma asianum* and *Penicillium oxalicum* were the species with the largest abundance (Table 1).

**TABLE 1:** Relative abundance of dominant fungi among samples at the genus level.

**Dominant Genus**	**LHL1-1(%)**	**LHL1-2(%)**	**LHL1-3(%)**	**NCQ1-1(%)**	**NCQ1-2(%)**	**NCQ1-3(%)**
*Lepidostroma*	97.78%	1.50%	2.83%	0.69%	0.37%	0.03%
*Acrostalagmus*	0.00%	0.00%	0.00%	0.01%	46.05%	0.01%
*Penicillium*	0.02%	2.63%	2.16%	0.44%	1.28%	28.63%
*Archaeorhizomyces*	0.00%	0.00%	28.44%	7.69%	0.06%	0.01%
*Simplicillium*	0.15%	3.63%	0.03%	0.02%	0.04%	1.45%
*Fusarium*	0.03%	0.71%	0.67%	0.15%	0.77%	3.51%
*Cryptococcus*	0.00%	0.00%	0.00%	0.00%	2.83%	0.03%
*Cladosporium*	0.14%	0.79%	0.15%	0.03%	2.10%	1.23%
*Aspergillus*	0.02%	0.05%	0.08%	0.03%	1.66%	0.22%
*Gibellulopsis*	0.00%	0.01%	0.04%	0.03%	0.15%	1.53%

In the sample of the South Wall, *Ascomycota* occupied a dominating position of phylum and accounted for 10.93–68.77% of reads of each sample, and the relevant mean abundance reached 47.50%. In different samples, the relative abundance at the genu level was significantly different. *Penicillium* was the most abundant genus in the South Wall sample, and accounted for 0.42–25.88% of the Tiger Ridge sample, with an average abundance of 9.09%. From the perspective of species, *Acrostalagmus luteoalbus*, *Penicillium oxalicum* were the most abundant species (Table 1). Assessment of sample overlapping was conducted to differentiate diversity shared by the 6 samples (Figures 7A,B). There were 177 bacterial operating taxonomic units (OTUs) in total, were shared in the samples (total abundance of 5712 OTUs). In addition, 6 samples shared 45 fungal OTUs from an overall fungal abundance of 324 OTUs. The outcome suggested that the microbial community of various sampling sites overlapped to a certain degree. This was especially distinctive for the fungal community, where *Fusarium* is the dominant genus, taking up a large proportion in all communities.

Evaluations of bacterial (XB, XN1, XN2) constituent parts (determined through sequencing of 16S rRNA V4 region) were performed with a sequence of amplicon through Illumina HiSeq2500 PE250 for specimens. The acquisition, with a total of 219934 reads of high quality, was achieved after removing READS of inferior quality, chimeras, trim adapter, bar codes and primer by filtration. Gene sequences of the entire16S rRNA were distributed to 36 bacterial phyla. A summary of ten taxa with the largest number at the level of phylum is shown in Table 2. Proteobacteria was phylum with the largest number, taking up 27.08–45.50% of each sample’s reads, and the relevant mean abundance reached 36.29%. *Bacteroidetes* were the second most numerous phylum, and the relevant mean abundance reached 14.22%. *Firmicutes* accounted for 20.00% of all reads in the sample XN.2, compared to only 2.78% to 12.08% in the remaining samples. Other major phyla included *Cyanobacteria* (6.07–11.47%, average of 9.60%), *Chloroflexi* (0.72–10.44%, average of 3.40%) and *Actinobacteria* (4.42–10.15%, average of 0.71%) (Figures 7C,D).

**TABLE 2:** Relative abundance of dominant bacterial species among samples.

**Dominant Genus**	**XB(%)**	**XN-1(%)**	**XN-2(%)**
*unidentified_Cyanobacteria*	11.27%	11.45%	6.00%
*Stenotrophomonas*	1.05%	0.20%	7.37%
*Nevskia*	0.09%	7.31%	1.43%
*Arenimonas*	0.05%	7.24%	0.72%
*Streptococcus*	0.14%	0.03%	6.37%
*Mucilaginibacter*	4.21%	0.66%	1.06%
*Methylotenera*	0.12%	2.80%	3.25%
*Acidothermus*	0.12%	3.12%	0.07%
*Pseudonocardia*	0.25%	2.12%	1.22%
*Lactococcus*	0.48%	2.03%	0.28%

### Dominant Fungal Identification and Isolation

**FIGURE 8:**
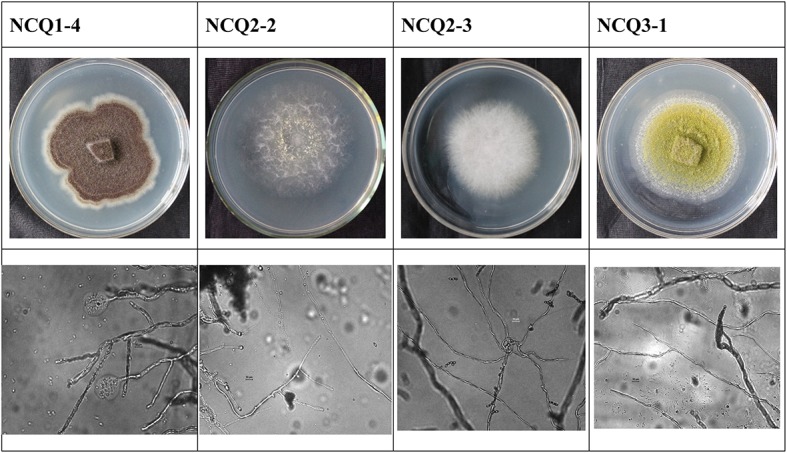
Colony and micromorphological features of four fungal isolate at 400x magnification.

The high-throughput sequencing results revealed dominance of *Lepidostroma* among the six samples, and we therefore attempted to isolate the dominant fungi to study their characteristics. Incubation on potato dextrose agar (PDA) plates at 28°C for 6 days resulted in the growth of some colonies. Nineteen strains were separated from the samples. We then sequenced the ITS1–5.8S rRNA-ITS2 gene regions of nineteen colonies (Table 3). *Penicillium oxalicum* (NCQ2-2), *Fusarium oxysporum* (NCQ2-3), *Aspergillus aculeatus* (NCQ1-4), and *Aspergillus nomius* (NCQ3-1) were separated by pure culturing (Figure 8), and the results were consistent with the high-throughput results, while *Lepidostroma*, which showed the highest content, was not cultured successfully.

**TABLE 3:** Molecular identification of strains isolated from the soil samples.

**Nucleotide Blast reference strains**					**Soil**					
**Fungi**	**Closet relative strain**	**Phylum**	**Accession number**	**Similarity (%)**	**NCQ-1**	**NCQ-2**	**NCQ-3**	**LHL-1**	**LHL-2**	**LHL-3**
NCQ1-1	*Penicillium* sp.	*Ascomycotina*	KM103311.1	99%	√	√	√	√	√	√
NCQ1-2	*Trichoderma* sp.	*Deuteromycotina*	KM203582.1	100%	√	√	√	√		
NCQ1-4	*Aspergillus aculeatus strain*	*Ascomycotina*	MH634496.1	100%	√	√	√	√	√	√
NCQ1-5	*Rhizomucor variabilis*	*Zygomycota*	KP067786.1	99%	√		√	√		√
NCQ1-6	*Mucor indicus*	*Zygomycota*	LC390229.1	100%	√				√	
NCQ1-7	*Talaromyces pinophilus*		MK041911.1	99%	√			√		
NCQ2-1	*Penicillium* sp.	*Ascomycotina*	KM103311.1	99%		√		√	√	√
NCQ2-2	*Penicillium oxalicum*	*Ascomycotina*	MH591400.1	100%	√	√	√	√	√	√
NCQ2-3	*Fusarium oxysporum*	*Ascomycota*	KT876657.1	100%	√	√	√	√	√	√
NCQ2-4	*Curvularia lunata*		KY404178.1	100%	√	√				
NCQ3-1	*Aspergillus nomius*	*Ascomycotina*	MF806072.1	100%	√	√	√	√	√	
NCQ3-2	*Trichoderma longibrachiatum*	*Deuteromycotina*	MH283936.1	99%			√			√
NCQ3-4	*Rhizomucor variabilis*	*Zygomycota*	EU616623.1	99%			√			
NCQ3-6	*Fungal endophyte* sp.		FJ613819.1	99%	√		√		√	√
LHL1-1	*Trichoderma ovalisporum*	*Deuteromycotina*	MF383149.1	99%	√			√		√
LHL1-2	*Rhizomucor variabilis*	*Zygomycota*	EU196747.1	99%	√			√		
LHL1-3	*Mucor irregularis*		MH862387.1	99%				√		
*LHL2-3*	*Penicillium janthinellum*	*Ascomycotina*	KU529846.1	99%	√				√	

### Protective Material Degradation Experiment

Internationally, some countries, such as Switzerland, Japan, the United States, and Italy, use epoxy resin as a reinforcing agent (Selwitz, 2011). In China, we also use epoxy resins frequently, so we chose epoxy resin as the experimental material in the present study. Fourier infrared spectroscopy is an analytical method for qualitatively and quantitatively analyzing the epoxy value of epoxy resin by using the characteristic absorption peak of the epoxy group in the near-infrared region. This method can not only easily analyze the epoxy group content of the epoxy resin matrix but can also analyze the functional structures and change the law of epoxy resin during curing (Wang et al., 2004).

Infrared spectroscopy usually divides the spectrum into a high-frequency region and a low-frequency region at 1500 CM^–1^. The former is the characteristic frequency region of chemical bonds and functional groups, and the absorption peak is minimal. The latter reflects the whole molecule due to vibration and rotation. The characteristic absorption peak, the infrared spectrum characteristic absorption peak of the microscopic component, is mainly distributed in the low-frequency region. According to the principles of infrared spectroscopy and organic chemistry, epoxy resin mainly includes epoxy groups. The infrared spectrum mainly exhibits absorption peaks: the characteristic peak of epoxy groups occurs at approximately 910 cm^–1^, and there are slight differences between different types of equipment and detection methods. In the figure, the left panel shows the results of the reaction performed for 15 days, the right panel shows the results of the reaction performed for 30 days, in the 15-day reaction, red indicates the control group (Supplementary Figure S1A), and the other three colors indicate the reaction groups. All of them exist in the ring of 910 cm-1 (Wang et al., 2015). The characteristic peak of oxygen was blue in the 30-day sample (Supplementary Figure S1B); the other three groups were the experimental group, and the control group exhibited the characteristic peak at 910 cm^–1^, while the characteristic peak of the experimental group at approximately 910 cm^–1^ was not as obvious. It can be observed that under the action of multiple strains, certain substances produced by bacteria and fungi may affect the properties of the epoxy resin itself, and the specific effects on the organic matter require further research.

### Soil Analysis Results With Protective Materials

**FIGURE 9:**
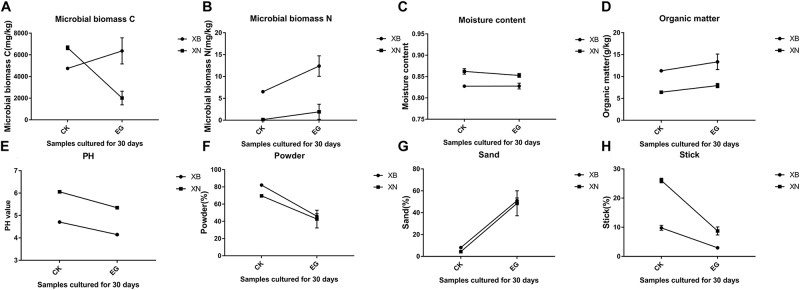
Analysis of the properties of soil containing ethyl orthosilicate. **(A)** content of microbial biomass C (mg/kg), **(B)** content of microbial biomass N (mg/kg), **(C)** percentage of moisture content, **(D)** content of organic matter, **(E)** PH, **(F)** percentage of powder, **(G)** percentage of sand, **(H)** percentage of sticks.

According to the literature, Portuguese Rodrigues et al. used tetraethyl orthosilicate and other materials in limestone and measured their penetration depth. The silicone reinforcing materials that have been widely used include alkyl silicate, ethyl silicate, siloxane and so on (Tarasov, 2001). For example, tetraethyl orthosilicate was widely used in the protection of stone cultural relics in 1936. However, the stress damage caused by the hydrophobicity and hydrophilicity of organosilicon is obvious, and this side effect will cause damage to cultural relics. Therefore, we performed this experiment to explore the effect at the Archaeological Ruins of Liangzhu City. According to the data in the figure, after 1 month of cultivation, the moisture contents of the two groups of soil samples containing tetraethyl orthosilicate did not change significantly compared with the control group, the PH value decreased slightly, and the organic matter content of the two groups was low. The amplitude increased, but the microbial biomass changed greatly, in which the amount of microorganisms in the XB group increased, and the amount of microorganisms in the XN group decreased. Compared with the sand, powder and stick contents of the control group, the percentages in the two groups of samples were significantly reduced, and the proportion of sand decreased. It can also be seen that the powder to stick ratio was decreasing. The amplitude was large. The grain size of the soil changed significantly compared to the control group. The sand, powder, and viscosity ratio decreased, and the soil became somewhat coagulated, while that in the control group was loose (Wilson and Wilson, 2014). In the experimental group, the ratio of microbial biomass C (Janzen, 2006), to N increased, and after the use of tetraethyl orthosilicate, the microorganisms became more abundant. Therefore, it was clear that tetraethyl orthosilicate has a great influence on the soil sites. If ethyl orthosilicate is used at earthen sites, the microbial biomass at the soil sites will increase, and the soil size will change, causing the soil to clot (Figure 9).

## Discussion

We conducted a preliminary investigation into the nature of soil microbial communities. At this moment, non-invasive sampling using adhesive tape strip was performed for subsequent microscopy and viability assays, as this approach offered the possibility of obtaining information about microbial colonization as well as the survival capability of microorganisms without causing harm to the surface. Six samples (NCQ-1, NCQ-2, NCQ-3, LHL-1, LHL-2, LHL-3) were characteristic of a marked presence of microorganisms, particularly fungi and algae. Epifluorescence images revealed that many fungal hyphae and spores and algal cells were active.

High-throughput sequencing of the fungal ITS1 region revealed that in the fungal assessment of the Liangzhu soil site, the genus *Liriodendron* was the most important fungus, followed by *Penicillium, Acrostalagmus*, and *Archaehorhizomyces.* The proportion of *Lepidostroma* in the Tiger Ridge (LHL) sample was 33.85%; the percentages of *Acrostalagmus* and *Penicillium* on the South City Wall (NCQ) were 16.24 and 9.10%, respectively. The fungi of the genus *Lepidostroma* are related to the growth of algae. In areas with more algae, the abundance of this fungus is higher. A morphological study of these fungi indicated that the thallus most closely resembled that of the central African species *Lepidostroma rugaramae*, as both species exhibit white-rimmed squamules with conspicuous maculae resulting from pyramidal photobiont columns. However, the basidiocarps are yellow to orange-brown, without the reddish tinge seen in *L. rugaramae*, and the apex is distinctively cream colored, especially when dry. Additionally, the spores are more elongated, making them larger overall, and the cells of the upper cortex of the thallus are polygonal in surface view and often multilayered (neither jigsaw-like in surface view nor consistently in a single layer as in *L. rugaramae* (Hodkinson and Smith, 2012; Yanaga et al., 2015). Because the Liangzhu soil environment is relatively humid, there are many types of fungi and algae present (Cutler et al., 2013). In the protection of earthen sites, it is recommended that soil sites are kept in a relatively dry state because a relatively dry environment can inhibit the growth of algae and fungi and can therefore inhibit the destruction caused by algae and fungi to a certain extent (Francesca and Claudia, 2008). However, it is also necessary to focus on the hazards of fungi in humid environments. From the results of the fungal analysis in the preserved soil, it was found that although the fungal community structure in each sample was different due to the geographical location, the main dominant genera were *Lepidostroma*, followed by *Penicillium, Acrostalagmus*, and *Archaehorhizomyces*. The extent of damage to these sites caused by these fungi requires further analysis. In initial ecosystems, cyanobacteria, algae, fungi, mosses and lichens are the first organisms to colonize a substrate, and these organisms represent a double-edged sword in relation to the soil. They may produce organic acids and other substances that damage environmental protection materials. On the other hand, they form a biological crust within the first millimeters of the surface. Furthermore, they can protect the soil and prevent soil erosion (Csotonyi et al., 2010; Wang et al., 2010; Seitz et al., 2014).

The protection of cultural heritage is increasingly important. One of the basic aspects of protection work is the protection of cultural relics in the later stages to avoid damaging them again. To identify a more effective protective material for cultural relics, organic materials have been studied over time. Organic protective materials range from natural to synthetic. Paraffin has a long history of being used to protect cultural relics. Its earliest recorded use was in 88 A.D. However, although paraffin can be used to protect cultural relics by dissolving the paraffin in organic solvents, its permeability is low. When the depth is not sufficient, it is easy for the paraffin to form a surface shell, and the surface will eventually peel off. Silicone materials have also been used as reinforcement and protection agents for stone cultural relics, mainly in Germany, Italy, Portugal, and the United States Portuguese Rodrigues et al. Ethyl orthosilicate and other materials have been used with limestone, and their penetration depth has been measured. The silicone reinforcing materials that have been widely used include alkyl silicate, ethyl silicate, siloxane and so on (Tarasov, 2001; Cardiano et al., 2003). For example, tetraethyl orthosilicate was widely used in the protection of stone cultural relics in 1936. However, the stress damage caused by the hydrophobicity of organosilicon and the hydrophilicity of stone is obvious, and this side effect will cause damage to cultural relics. Polymers that are widely used include acrylic resin, epoxy resin and so on. Epoxy resin was used for grouting treatment of Grotto 6 at the Yungang Grottoes (Huang, 2003). Polysiloxane was used to reinforce Han Baiyu at the Palace Museum (Hai-Ping et al., 2010). Acrylic resin was used to reinforce sculpture 71 on the Siena Cathedral gate and has also been used to reinforce sandstone relics. Materials such as organic fluorine polymers have also been used for protection (Yasumoto et al., 1998). These materials have achieved different results in the restoration and reinforcement of different stone cultural relics, but they also have some shortcomings, such as low and poor permeability, and a limited service life, along with an inexpensive price. It is easy for microorganisms to multiply on protective polymer materials. In 2005–2008, the Committee for Conservation of the International Council of Museums Modem carried out some research on microbial degradation of protective polymer materials. Many protective polymer materials such as epoxy resin and tetraethyl orthosilicate are needed for later-stage protection. Epoxy resin presents excellent physical, mechanical and electrical insulation properties, bonding properties with various materials, and flexibility in its application, which other thermosetting plastics do not exhibit. Therefore, it can be made into coatings, composite materials, castables, adhesives, molding materials and injection molding materials, which are commonly used in site protection. Ethyl orthosilicate is a colorless liquid that is mainly used as a heat-proof coating, chemical-resistant coating and organic synthesis intermediate.

Based on the previous examples, we consulted additional literature. We preliminarily tested the degradability of epoxy resin. The humid environment at the location of the Archaeological Ruins of Liangzhu City results in abundant microorganisms. During the time when epoxy resin is in use, the resin will encounter a large number of fungi. We simulated this environment and sped up this reaction in the laboratory. The above experiments revealed the presence of fungi and bacteria. These organisms will react with epoxy resin, causing some changes in the resin and impacting their own characteristics. As a widely used protective material, epoxy resin is not entirely unaffected by the environment in the process of its use, although the effect of this impact is currently unknown. However, we should pay attention to such effects, which have a certain reference significance for future protection work. In our next study, we can explore the reaction mechanism involved. To improve the functioning of the protective materials in the protection of sites and the use of protective materials, we should also ensure that they have less of an impact on the environment. Ethyl orthosilicate is a colorless liquid that is mainly used as a heat-proof, chemical-resistant coating and an organic synthesis intermediate. Compared with epoxy resin, tetraethyl orthosilicate is easier to sprinkle on the soil during use, so we chose sites of orthosilicate use to test its impact on soil properties. Through the above experiments, we found that tetraethyl orthosilicate has a great impact on the environment. As earthen sites, the Liangzhu cultural ruins contain a great deal of soil. After the use of tetraethyl orthosilicate in the experiment, the soil particle size changed greatly. The stick and powder contents of the soil decreased to a great extent, that of sand increased to a certain extent, the contents of small and medium particulate matter in the soil decreased, that of large particulate matter increased, and the soil began to coagulate. This state will affect the survival of microorganisms in the soil. If the ethyl orthosilicate is used at an earthen site, the microbial biomass of the soil at the site will increase, and soil in this state will cause intensification of soil weathering, facilitating the loss of soil and water, and ultimately destroying the ecological environment of the entire site, causing irreversible losses (Gao et al., 2014).

## Data Availability Statement

The datasets generated for this study can be found in the Nucleotide Sequence Accession Number The nucleotide sequences of strains have been deposited in the DDBJ/GenBank/EMBL database under the accession numbers MN509056-MN509073 for fungal ITS sequences. The raw sequencing data could be download-ed at the NCBI Sequence Read Archive (SRA) with the study accession number PRJNA573470.

## Author Contributions

JP and MS conceived and designed the study. MS, FZ, XH, YH, and LS performed the experiments. NJ, BC, MK, and QG provided assistance during the experiments. MS analyzed the data and wrote the manuscript. JP reviewed and edited the manuscript. All authors read and approved the manuscript.

## Conflict of Interest

The authors declare that the research was conducted in the absence of any commercial or financial relationships that could be construed as a potential conflict of interest.

## Supplementary Material

FIGURE S1 **(A)** The red spectrum represents the control group, the other colors are the three experimental groups, and the position of the red circle is 910 cm^–1^. **(B)** The green spectrum represents the control group, the other colors are three experimental groups, and the position of the red circle is 910 cm^–1^.
