# Supplementary figures and images for: Analysis of Microbial Community in the Archaeological Ruins of Liangzhu City and Study on Protective Materials
Source: Front Microbiol. 2020 Apr 15;11:684. doi: 10.3389/fmicb.2020.00684 (PMC7174599; doi:10.3389/fmicb.2020.00684)

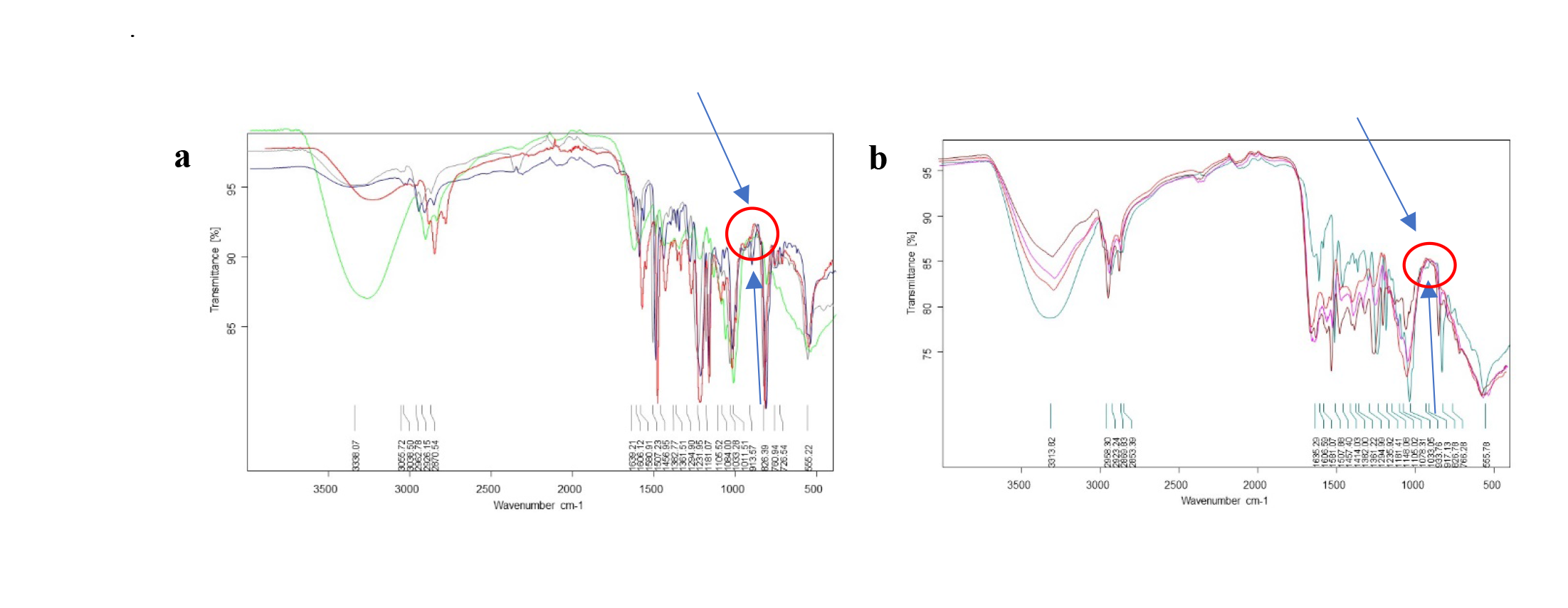

Supplement: FIGURE S1 — (A) The red spectrum represents the control group, the other colors are the three experimental groups, and the position of the red circle is 910 cm–1. (B) The green spectrum represents the control group, the other colors are three experimental groups, and the position of the red circle is 910 cm–1. [file Image_1.TIF]
